# Real-life effectiveness of COVID-19 vaccine during the Omicron variant-dominant pandemic: how many booster doses do we need?

**DOI:** 10.1080/22221751.2023.2174779

**Published:** 2023-02-15

**Authors:** Paskorn Sritipsukho, Thana Khawcharoenporn, Boonying Siribumrungwong, Pansachee Damronglerd, Nuntra Suwantarat, Araya Satdhabudha, Chanapai Chaiyakulsil, Phakatip Sinlapamongkolkul, Auchara Tangsathapornpong, Pornumpa Bunjoungmanee, Sira Nanthapisal, Chamnan Tanprasertkul, Naiyana Sritipsukho, Chatchai Mingmalairak, Anucha Apisarnthanarak, Pichaya Tantiyavarong

**Affiliations:** a Center of Excellence in Applied Epidemiology, Thammasat University, Pathumthani, Thailand; b Department of Pediatrics, Faculty of Medicine, Thammasat University, Pathumthani, Thailand; c Department of Internal Medicine, Faculty of Medicine, Thammasat University, Pathumthani, Thailand; d Department of Surgery, Faculty of Medicine, Thammasat University, Pathumthani, Thailand; e Department of Internal Medicine, Chulabhorn International College of Medicine, Thammasat University, Pathumthani, Thailand; f Department of Obstetrics & Gynecology, Faculty of Medicine, Thammasat University, Pathumthani, Thailand; g Thammasat Postdoctoral Fellowship, Thammasat University, Pathumthani, Thailand; h Department of Clinical Epidemiology, Faculty of Medicine, Thammasat University, Pathumthani, Thailand

**Keywords:** COVID-19, real-life, vaccine, effectiveness, Omicron variant, Thailand

## Abstract

The surge in coronavirus disease 2019 (COVID-19) caused by the Omicron variants of the severe acute respiratory syndrome coronavirus 2 necessitates researches to inform vaccine effectiveness (VE) and other preventive measures to halt the pandemic. A test-negative case–control study was conducted among adults (age ≥18 years) who were at-risk for COVID-19 and presented for nasopharyngeal real-time polymerase chain reaction testing during the Omicron variant-dominant period in Thailand (1 January 2022–15 June 2022). All participants were prospectively followed up for COVID-19 development for 14 days after the enrolment. Vaccine effectiveness was estimated and adjusted for characteristics associated with COVID-19. Of the 7971 included individuals, there were 3104 cases and 4867 controls. The adjusted VE among persons receiving 2-dose, 3-dose, and 4-dose vaccine regimens for preventing infection and preventing moderate-to-critical diseases were 33%, 48%, 62% and 60%, 74%, 76%, respectively. The VE were generally higher among those receiving the last dose of vaccine within 90 days compared to those receiving the last dose more than 90 days prior to the enrolment. The highest VE were observed in individuals receiving the 4-dose regimen, CoronaVac-CoronaVac-ChAdOx1 nCoV-19-BNT162b2 for both preventing infection (65%) and preventing moderate-to-critical diseases (82%). Our study demonstrated increased VE along with an increase in number of vaccine doses received. Current vaccination programmes should focus on reducing COVID-19 severity and mandate at least one booster dose. The heterologous boosters with viral vector and mRNA vaccines were highly effective and can be used in individuals who previously received the primary series of inactivated vaccine.

## Introduction

The evolution of severe acute respiratory syndrome coronavirus 2 (SARS-CoV-2) has caused the emergence of its various variants associated with multiple epidemic waves of coronavirus diseases 2019 (COVID-19). The Omicron variant (B.1.1.529) of SARS-CoV-2 is the latest variant responsible for the surge in COVID-19 worldwide with sublineages BA.1 and BA.2 being dominant at the beginning of the pandemic in 2022. In Thailand, the Omicron variant has contributed to the fourth wave of COVID-19 epidemic and the significantly higher number of infected cases than the previous epidemic waves [1]. Compared to other variants of SARS-CoV-2, the Omicron variant has been reported to transmit more rapidly but cause less severe diseases and contribute to lower rates of COVID-19-associted hospitalization and mortality [2–5]. Nonetheless, the high rates of intensive care requirement and in-hospital mortality were still observed among unvaccinated individuals and in those with advanced age, obesity, uncontrolled diabetes, immunocompromised status, and chronic respiratory, cardiovascular, liver and renal diseases [2,3]. These burdens underscore the need for interventions to impede the global pandemic associated with the Omicron variant.

During the previous epidemic waves caused by Alpha, Beta, and Gamma variants of SARS-CoV-2, vaccination with primary series of inactivated viral, viral vector, and mRNA vaccines had moderate to high effectiveness in preventing symptomatic infections [6,7], while during the Delta variant pandemic, either a heterologous or homologous booster with viral vector or mRNA vaccines were required to achieve high effectiveness [7,8]. However, during the Omicron variant pandemic, concerns have been raised in regard to the Omicron variant’s mutations resulting in changes in the spike protein, which enable immune escape and lead to decreased vaccine and monoclonal antibody effectiveness [9]. Recent studies have been mostly conducted on vaccine effectiveness (VE) against the Omicron variant of mRNA vaccines [2,10–15]. The reported VE of two-dose and three-dose mRNA vaccine regimens were 47–62% and 44–70% for preventing infection and 65–80% and 70–94% for preventing severity, respectively. Nonetheless, these VE decreased significantly to below 50% after 90 days [12,14,15]. For inactivated viral and viral vector vaccines, there were limited data on the VE when used as the primary series against the Omicron variant. The reported VE for the two-dose CoronaVac were 25% for preventing infection and 58–92% for preventing severity [16,17], while the reported VE for the two-dose ChAdOx1 nCoV-19 were 2–49% for preventing infection and 32–66% for preventing severity [18,19]. Only two studies demonstrated the VE of 59–68% for preventing infection and 84% for preventing severity by adding a booster dose of mRNA vaccines to the primary series of these vaccines [19,20].

In early 2022, the uncertainty about how long the COVID-19 pandemic will last, possibility of decreased VE against the Omicron variant and the decline in immunity overtime had led to the decision to receive the second booster dose among Thai population, especially those at-risk for severe diseases and mortality. Nonetheless, data existing on the real-life VE against the Omicron variant among these various vaccine regimens with differences in type and number of booster dose are currently limited.

## Methods

### Study design and setting

A test-negative case–control study was conducted among COVID-19 at-risk individuals at Thammasat University Hospital (TUH) and Thammasat Field Hospital (TFH) in Pathumthani, Thailand. The TUH is a 700-bed tertiary-care academic medical facility that serves population in northern Bangkok and central Thailand while TFH is a 490-bed field hospital branch of TUH, established specifically to provide comprehensive care to asymptomatic and mild COVID-19 patients. This study was conducted during the period from 1 January 2022 to 15 June 2022 when the Omicron variant was a dominant variant causing COVID-19 in this region and in Thailand [1]. The test-negative case–control study design has advantages in assessment of the VE since it allows comparison of the VE between various vaccine dosing and regimens in the same platform and provides timely results during the ongoing pandemic [21]. The mass COVID-19 vaccination programmes have been initiated by the Thai Government and Ministry of Public Health (MOPH) since March 2021. The available primary series vaccine regimens during the initial period mainly included two-dose CoronaVac (4 weeks apart between the two doses) and two-dose ChAdOx1 nCoV-19 (12 weeks apart between the two doses), while two-dose BNT162b2 (4 weeks apart between the two doses) had become available later. The booster doses of the vaccines have been available in the latter half of the year 2021. These included one dose of ChAdOx1 nCoV-19 or one to two doses of BNT162b2 given at 3–6 months after the primary series or after the last dose of the previous vaccines. This study was approved by the Human Research Ethics Committee of Thammasat University (Medicine).

### Study population

The study population included adults ≥18 years old at-risk for COVID-19 who presented TUH or TFH, met the criteria for patients under investigation (PUI) for COVID-19 (Supplementary Table) and received nasopharyngeal RT–PCR test for SARS-CoV-2 at either facility. All eligible individuals were approached and asked for consent to participate in the study. The exclusion criteria were those who had prior COVID-19 and/or did not complete the 14-day follow-up period to assess COVID-19 development.

### Study protocols and definitions

At TUH and TFH, the screening and management of COVID-19 were according to the country’s national guidelines. Baseline nasopharyngeal RT–PCR tests were performed in all PUI within 3–5 days after COVID-19 contact or having symptoms consistent with COVID-19. The timeline of the test was based on the incubation period of the Omicron variant reported previously [22]. All of the PUI underwent subsequent nasopharyngeal RT–PCR tests if they later developed symptoms or had the initial test less than 3 days after COVID-19 contacts. The repeated RT–PCR test was to ensure that asymptomatic COVID-19 cases were not missed. For assessment of VE for COVID-19 prevention, cases were defined as PUI whose initial or subsequent nasopharyngeal RT–PCR tests were positive while controls were those with negative initial and all follow-up RT–PCR tests during the 14-day period. All cases were categorized into asymptomatic, mild, moderate, severe, and critical diseases according to the World Health Organization criteria [23]. All controls were prospectively followed for COVID-19 development via Chatbot software and telephone interview. For assessment of VE for preventing moderate to critical diseases, cases were defined as those who had moderate, severe, or critical illnesses, while controls were those with negative all RT–PCR tests throughout the 14-day follow-up period excluding those with asymptomatic and mild diseases. This definition of controls would allow for assessing the true VE for preventing moderate to critical diseases, compared to no infection. Completion of each dose of vaccination was defined as receipt of the first, second, third, and fourth doses of the vaccines for at least 21, 14, 7, and 7 days at the time of enrolment, respectively [24–26].

Data including demographics, comorbidities, COVID-19 contact, and history of COVID-19 vaccination were collected using Research Electronic Data Capture (REDCap). In this study, we randomly tested for causative variants of SARS-CoV-2 in one of every 9 participants who gave a consent. The primary outcomes were the VE in preventing COVID-19 in regards to the number of doses and type of vaccine received, while the secondary outcomes were the VE in preventing moderate to critical COVID-19 in regards to the number of dose and type of vaccine received and numbers of cases stratified according to disease severity and vaccine status.

### Statistical analysis

All analyses were performed using STATA, version 14 (StataCorp, College Station, Texas). Categorical data were compared between cases and controls using Chi-square test or Fisher’s exact test as appropriate. Based on the reported average VE of 70% against Omicron variant infection and VE of 90% against severity for the three-dose vaccine regimens in the previous study [27,28] and the expected vaccination coverage of 50% for the major vaccine types received by Thai population, the required sample sizes to have a precision rate of ±10% and a 5% type I error rate were 346 for cases and 346 for controls to determine VE for preventing infection and were 80 for cases and 80 for controls to determine VE for preventing severity [29]. To determine the VE, we first estimated the odds of COVID-19 cases and the odds of moderate to critical cases with 95% confidence interval (CI) among vaccinated individuals compared with unvaccinated individuals. We then calculated VE using the formula (1-Odds ratio) × 100%. By multivariable regression analysis, the calculated VE with 95% CI were adjusted for covariates associated with COVID-19, which had *P* < 0.05 from the univariable analysis.

## Results

### Characteristics of the study population

There were 7971 individuals who had completed the 14-day follow-up period. Among the 7971 included individuals, 3104 (38.9%) were cases and 4867 (61.1%) were controls. None of these individuals had prior COVID-19. Demographics and characteristics of the included study population are shown in Table 1. Compared to controls, cases were significantly older, had higher proportion of being female and unemployed, and had lower education levels. Higher proportion of cases had at-risk comorbidities and higher numbers of persons with COVID-19 at home compared to controls. By multivariable logistic regression analysis, age, sex, education level, being unemployed, having any at-risk comorbidities, and having persons with COVID-19 at home were independent factors associated with COVID-19.

**Table 1.** Characteristics of the study cohort, cases and controls.

Characteristic	All (*N* = 7971)	Cases (*N* = 3104)	Controls (*N* = 4867)	*P*-value^a^
Age (years)				<0.001
18–60	7272 (91.2)	2566 (82.7)	4706 (96.7)	
>60	699 (8.8)	538 (17.3)	161 (3.3)	
Sex				<0.001
Male	2746 (34.5)	1251(40.3)	1495 (30.7)	
Female	5225 (65.5)	1853 (59.7)	3372 (69.3)	
Highest education				<0.001
Primary school or less	763 (9.6)	538 (17.3)	225 (4.6)	
Secondary school	2224 (27.9)	916 (29.5)	1308 (26.9)	
Bachelor degree	4321 (54.2)	1360 (43.8)	2961 (60.8)	
More than Bachelor degree	515 (6.5)	163 (5.3)	352 (7.2)	
Undetermined	148 (1.9)	127 (4.1)	21 (0.4)	
Occupation				<0.001
Healthcare worker	2003 (25.1)	745 (24)	1258 (25.9)	
Office worker	2486 (31.2)	460 (14.8)	2026 (41.6)	
Self-employed business	1708 (21.4)	730 (23.5)	978 (20.1)	
School or college student	477 (6.0)	258 (8.3)	219 (4.5)	
Unemployed	1297 (16.3)	911 (29.4)	386 (7.9)	
Comorbidities				
Obesity (BMI > 27 kg/m^2^)	1700 (21.3)	774 (24.9)	926 (19.0)	<0.001
Cardiovascular diseases	995 (12.5)	663 (21.4)	332 (6.8)	<0.001
Diabetes mellitus	523 (6.6)	359 (11.6)	164 (3.4)	<0.001
Chronic lung/airway diseases	243 (3.1)	118 (3.8)	125 (2.6)	0.002
Chronic kidney diseases	139 (1.7)	110 (3.5)	29 (0.6)	<0.001
Cerebrovascular diseases	129 (1.6)	96 (3.1)	33 (0.7)	<0.001
Malignancy	103 (1.3)	67 (2.2)	36 (0.7)	<0.001
Having persons with COVID-19 at home				<0.001
None	5745 (72.1)	1958 (63.1)	3787 (77.8)	
1 person	1361 (17.1)	597 (19.2)	764 (15.7)	
≥2 persons	654 (8.2)	391 (12.6)	263 (5.4)	
Undetermined	211 (2.6)	158 (5.1)	53 (1.1)	

Note: Data are in numbers (%). BMI = body mass index; COVID-19 = coronavirus disease 2019.

^a^ Comparison between cases and controls.

During the 14-day follow-up period, 146 of 5013 individuals with initial negative RT–PCR test had positive test results on the repeated RT–PCR testing. Of the 3104 cases, 111 (3.6%), 2566 (82.7%), 310 (10%), 81 (2.6%), and 36 (1.2%) had asymptomatic, mild, moderate, severe, and critical diseases, respectively. During the study period, the Omicron variant accounted for 97–100% of viruses causing COVID-19 in this region corresponding with the result of our random variant testing among the 363 participating individuals that revealed Omicron as a major causative variant (97.8%;) (sublineages BA.1 64.8% and BA.2 35.2%).

### Vaccine effectiveness by the number of doses

**Figure 1.**
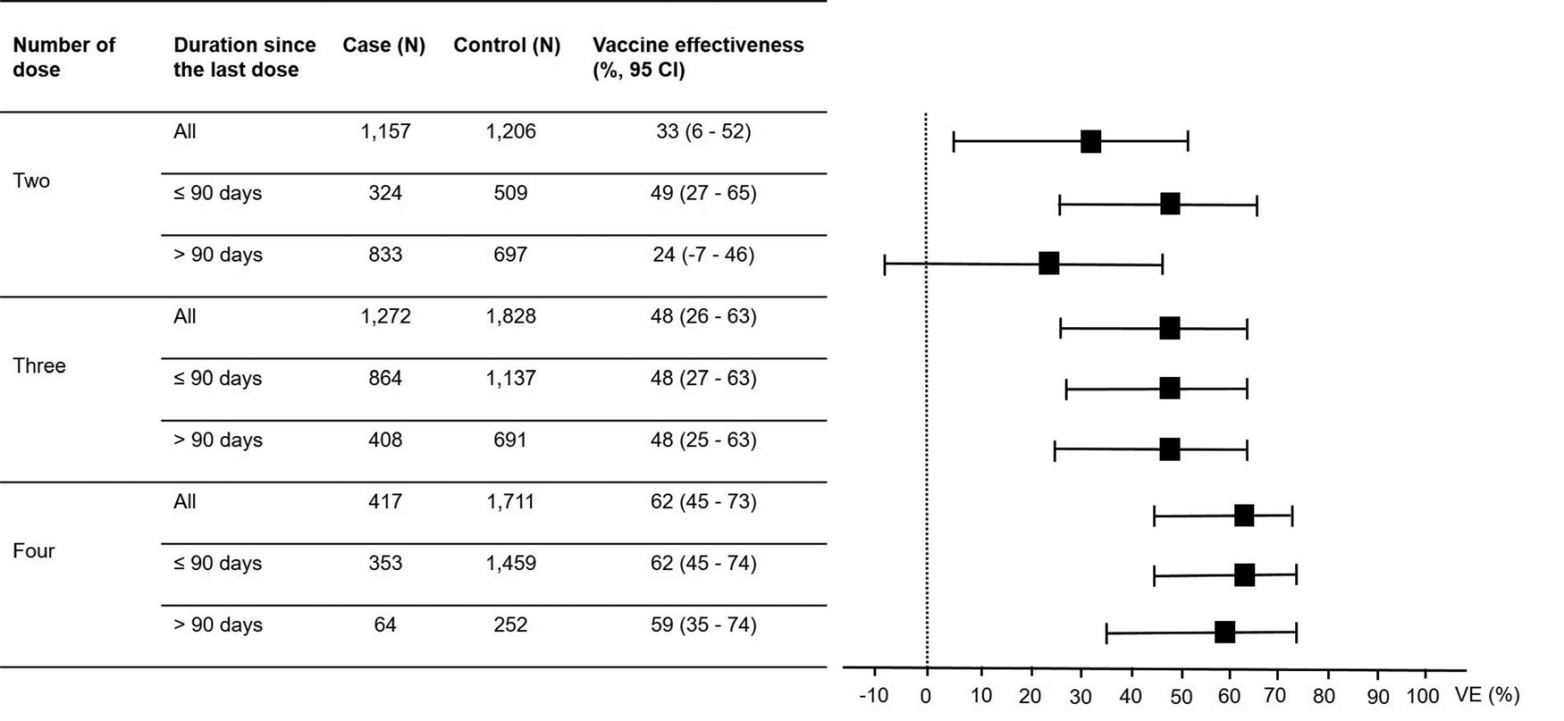
Vaccine effectiveness (VE) for preventing Omicron variant-associated infection according to number of dose received by the study participants. Note: The VE were adjusted for age, sex, education level, being unemployed, having any at-risk comorbidities and having persons with COVID-19 at home. I bars indicate 95% confidence intervals (CI).

**Figure 2.**
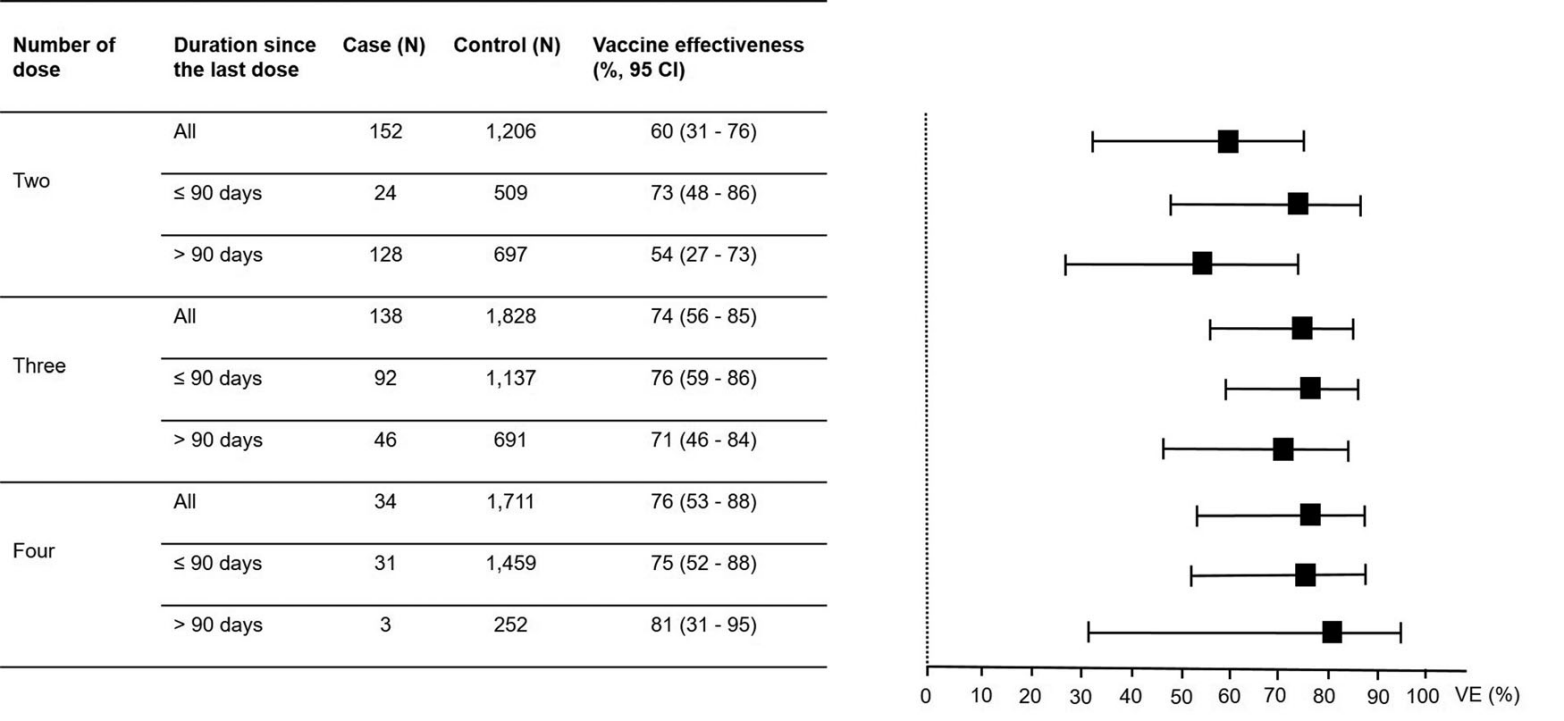
Vaccine effectiveness (VE) for preventing Omicron variant-associated moderate to critical diseases according to number of dose received by the study participants. Note: The VE were adjusted for age, sex, education level, being unemployed, having any at-risk comorbidities and having persons with COVID-19 at home. I bars indicate 95% confidence intervals (CI). Cases were those with moderate to critical diseases and controls were those without COVID-19.

Of the 7971 individuals participating in this study, 7688 (96.5%) had received at least one dose of COVID-19 vaccines, of which 90 (1.2%), 2363 (30.7%), 3100 (40.3%), 2128 (27.7%), and 7 (0.01%) had received one, two, three, four, and five doses of the vaccines, respectively. Given the small number of individuals who had received one and five doses of the vaccines, further analyses were focused only on individuals who had received two, three, and four doses of the vaccines. The VE reported in this study were adjusted for all independent factors associated with COVID-19. In the analysis for “any vaccine” for COVID-19 prevention (Figure 1), the VE increased from 33% (95% CI, 6–52) for the two-dose regimens to 48% (95% CI, 26–63) for the three-dose regimens and to 62% (95% CI, 45–73) for the four-dose regimens. Comparing between those receiving the last dose of the vaccines within 90 days and more than 90 days at the time of enrolment, the VE for those receiving the last dose of the vaccines within 90 days were higher for any two-, three- and four-dose regimens (Figure 1). In the analysis for “any vaccine” for preventing moderate to critical diseases (Figure 2), the VE increased from 60% (95% CI, 31–76) for the two-dose regimens to 74% (95% CI, 56–85) for the three-dose regimens and to 76% (95% CI, 53–88) for the four-dose regimens. The VE for preventing moderate to critical diseases were higher among those receiving the last dose of the vaccines within 90 days compared to those receiving the last dose more than 90 days at the time of enrolment for two- and three-dose regimens (Figure 2).

### Vaccine effectiveness by the types vaccines

For the two-dose vaccine regimens, the three most common regimens received by the 2363 individuals were two-dose ChAdOx1 nCoV-19 (*N* = 1209, 51.2%), two-dose CoronaVac (*N* = 397, 16.8%), and two-dose BNT162b2 (*N* = 293, 12.4%). For the three-dose regimens, the three most common regimens received by the 3100 individuals were two-dose ChAdOx1 nCoV-19 plus one BNT162b2 booster (*N* = 1387, 44.7%), two-dose CoronaVac plus one BNT162b2 booster (*N* = 960, 31%), and two-dose CoronaVac plus one ChAdOx1 nCoV-19 booster (*N* = 480, 15.5%). For the four-dose regimens, the two most common regimens received by the 2128 individuals at enrolment were two-dose CoronaVac plus two BNT162b2 boosters (*N* = 1202, 56.5%) and two-dose CoronaVac plus one ChAdOx1 nCoV-19 booster and one BNT162b2 booster (*N* = 836, 39.3%). All of these regimens were further analysed for VE that were adjusted for all independent factors associated with COVID-19 (Table 2). For COVID-19 prevention, the VE among individuals receiving two-dose CoronaVac plus one ChAdOx1 nCoV-19 booster and one BNT162b2 booster was highest (65%, 95% CI 49–76), followed by those receiving two-dose CoronaVac plus two BNT162b2 boosters (60%, 95% CI 42–73), and those receiving two-dose CoronaVac plus one BNT162b2 booster (56%, 95% CI 37–70). The VE among those receiving the last dose of the vaccines within 90 days before enrolment were mostly higher than those receiving the last dose more than 90 days before enrolment. For preventing moderate to critical diseases, the VE among individuals receiving two-dose CoronaVac plus one ChAdOx1 nCoV-19 booster and one BNT162b2 booster was highest (82%, 95% CI 59–92), followed by those receiving two-dose CoronaVac plus one BNT162b2 booster (76%, 95% CI 52–88), and those receiving two-dose CoronaVac plus one ChAdOx1 nCoV-19 booster (69%, 95% CI 36–85). Due to the small sample size of individuals with moderate to critical diseases, the VE for each type of vaccine regimen could not be analysed for subgroups receiving the last dose of the vaccine within 90 days or more than 90 days before enrolment.

**Table 2.** Vaccine effectiveness for preventing infection and severity of COVID-19 among various vaccine regimens.

No. of dose	Vaccine regimen	Duration since the last dose	Control (*N*)	Preventing infection		Preventing moderate to critical disease	
				Case (*N*)	Vaccine effectiveness (95% CI)^a^	Case (*N*)	Vaccine effectiveness (95% CI)^a^
2	CoronaVac-CoronaVac	All	193	204	26 (−9–50)	32	−4 (−50–45)
		≤90 days	49	33	47 (7–70)	NA	
		>90 days	144	171	21 (−15-47)	NA	
	ChAdOx1- ChAdOx1	All	601	608	38 (13–56)	90	56 (25–74)
		≤90 days	187	104	61 (41–74)	NA	
		>90 days	414	504	30 (0–51)	NA	
	BNT162b2- BNT162b2	All	154	139	23 (−13–49)	15	30 (−37–69)
		≤90 days	122	89	38 (4-60)	NA	
		>90 days	31	50	−38 (−60–24)	NA	
3	CoronaVac-CoronaVac- BNT162b2	All	653	307	56 (37–70)	17	76 (52–88)
		≤90 days	349	185	60 (42–73)	NA	
		>90 days	304	122	51 (27–67)	NA	
	ChAdOx1- ChAdOx1- BNT162b2	All	713	674	39 (13–57)	98	58 (28–75)
		≤90 days	587	548	41 (16–58)	NA	
		>90 days	126	126	26 (−12–52)	NA	
	CoronaVac-CoronaVac- ChAdOx1	All	300	180	53 (31–68)	17	69 (36–85)
		≤ 90 days	55	38	50 (15–71)	NA	
		>90 days	245	142	54 (31–69)	NA	
4	CoronaVac-CoronaVac- BNT162b2-BNT162b2	All	984	218	60 (42–73)	19	57 (7–80)
		≤90 days	862	195	58 (36–71)	NA	
		>90 days	122	23	65 (36–80)	NA	
	CoronaVac-CoronaVac- ChAdOx1-BNT162b2	All	667	169	65 (49–76)	9	82 (59–92)
		≤90 days	545	129	67 (51–78)	NA	
		>90 days	122	40	49 (15–70)	NA	

Note: CI = confidence interval; NA = not analyzable due to too small sample size in case and control groups.

^a^ Adjusted for age, sex, educational level, being healthcare worker, and having any comorbidities at-risk for disease progression.

The distribution of individuals receiving each specific vaccine regimen stratified according to disease severity is shown in Table 3. Among the individuals receiving the four-dose regimens, none had severe or critical illnesses.

**Table 3.** COVID-19 vaccination status of the study participants stratified according to diseases status and severity.

Vaccination status/Vaccine regimen	Cases/Disease severity				
	Asymptomatic	Mild	Moderate	Severe	Critical
Unvaccinated	12	99	44	28	24
2 doses					
CoronaVac-CoronaVac	9	163	30	1	1
ChAdOx1-ChAdOx1	26	492	65	21	4
BNT162b2-BNT162b2	3	121	12	2	1
3 doses					
CoronaVac-CoronaVac-BNT162b2	12	279	16	1	0
ChAdOx1- ChAdOx1-BNT162b2	23	553	78	16	4
CoronaVac-CoronaVac-ChAdOx1	4	159	14	2	1
4 doses					
CoronaVac-CoronaVac-BNT162b2-BNT162b2	6	193	19	0	0
CoronaVac-CoronaVac-ChAdOx1-BNT162b2	5	154	9	0	0

## Discussion

Our study evaluated the real-life VE of COVID-19 vaccines against the Omicron variant in the unique setting where most of the population had received the primary series of inactivated viral and viral vector vaccines with or without 1–2 heterologous booster doses of a viral vector or an mRNA vaccine. Given the timeline of the Omicron variant-dominant epidemic in the country, the analyses focused on the two-, three- and four-dose vaccine regimens which most of the Thai population had already received at the time of enrolment. The study findings demonstrated the increased VE in accordance with the increase in number of received vaccine dose, however even the 4-dose regimens (the highest number of dose) only achieved moderate level of VE against the Omicron variant infection. These findings were consistent with previous studies that showed the higher VE of three-dose homologous inactivated viral, viral vector, and mRNA vaccine regimens than the VE of two-dose homologous regimens of each type of the vaccines [10,16,18,30,31] and the higher VE of a four-dose mRNA vaccine regimen than a three-dose mRNA vaccine regimen for preventing COVID-19 [32,33]. These higher VE of the three- and four-dose regimens are likely due to the higher level of antibody and/or memory T-cells generated by the first and second booster doses given after the primary series vaccines [34].

To evaluate the immune sustainability against Omicron variant infections, we compared the VE between those receiving the last vaccine dose within 90 days vs. more than 90 days at the time of enrolment. We found that the VE when the last dose received for more than 90 days was significantly lower than the VE when the last dose received within 90 days for the two-dose vaccine regimens. However, the VE of these two different durations since the last dose were comparable for the three- and four-dose regimens. The more durable VE overtime of the three- and four-dose regimens were consistent with the findings from other studies [12,14,19,35] and most likely due to the slower waning of the IgG level after the 3rd and 4th doses compared to after the 2nd dose or the primary series [36]. However, the VE mostly dropped significantly to less than 50% regardless of the dosing regimen if the last dose was received for more than 6 months [19].

Although the VE for preventing infection were low to moderate against the Omicron variant, our study demonstrated the higher VE for preventing moderate to critical diseases. We found that the VE for preventing severity were significantly higher when receiving 1–2 booster doses (3^rd^ and/or 4^th^ doses) than when receiving only the primary series or two-dose regimens. These findings were similar to those reported from the studies of an mRNA vaccine [2,11,14,17], a viral vector vaccine [18], and an inactivated vaccine [16,17]. In regards to the immune sustainability, the VE of the three- and four-dose regimens were comparable between those receiving the last dose within 90 days vs. more than 90 days, while the VE of the two-dose regimens were significantly higher for those receiving the last dose within 90 days than those receiving the last dose more than 90 days for preventing severity. These findings indicated the more durable VE for preventing severity in those receiving 1–2 booster doses after the primary series, similar to the VE results for preventing infection. However, the slightly lower VE in preventing moderate to critical diseases among those receiving the last dose of the vaccines within 90 days compared to those receiving the last dose more than 90 days for the four-dose regimens, which was inconsistent with the findings for the two-dose and three-dose regimens, was likely due to the limited sample size of cases among those receiving the four-dose regimens.

We further analysed the VE according to the type of the primary series with and without booster vaccines. For preventing infection, the VE for the primary series (2 doses) of CoronaVac, ChAdOx1 nCoV-19, and BNT162b2 were generally lower than 50%. These results were consistent with the VE reported from the previous studies [14,16,18,19,30] and suggested inadequate protection against the Omicron variant infection. Among those who received one booster dose (3rd dose) with either ChAdOx1 nCoV-19 or BNT162b2, the highest VE for preventing infection (56%) and preventing moderate to critical diseases (76%) were observed in those receiving CoronaVac-CoronaVac- BNT162b2. The increased VE of this heterologous booster regimen may be explained by the increase in neutralizing antibody level against the Omicron variant demonstrated in another study [37]. To the best of our knowledge, this study is the first to describe the real-life VE against the Omicron variant of the 4-dose regimens that used heterologous boosters. We found that the CoronaVac-CoronaVac- BNT162b2-BNT162b2 and CoronaVac-CoronaVac- ChAdOx1 nCoV-19-BNT162b2 had comparable VE for preventing infection, however, the latter regimen had higher VE for preventing severity. This highest VE of this heterologous 4-dose regimen could be due to the greatest induction of neutralizing antibody as described in a previous Thai study [38]. Altogether, our study results suggested the utility of the heterologous three- and four-dose regimens for preventing Omicron variant-associated infection and severity, especially in settings where the primary series vaccines were inactivated vaccines.

The strengths of the study included the test-negative case–control design which allows for comparing of VE of various regimens within the same platform while the confounders from healthcare-seeking behaviours could be minimized. The prospective cohort study design for data collection facilitated accurate obtaining baseline and follow-up variables and minimizing missing data and misclassification biases. In addition, detailed information of COVID-19 vaccine, including type, number of dose, and time of vaccine exposure were derived and validated from the national vaccine database. However, some limitations should be noted. First, given that the study was conducted in a single centre, generalizability may be limited to other settings or to population with no or limited healthcare access. Second, the causative variants of SARS-CoV-2 were not determined in each individual infected case. Nonetheless, the results of our random variant testing and the MOPH SARS-CoV-2 variant surveillance report suggested that the Omicron variant was dominant during the study period. Third, although the different VE may be subjected to the duration from the last vaccine dose received to the time of study enrolment, we minimized this effect by categorizing the time to within or more than 90 days since the last dose for better comparison of the VE. Fourth, the VE of each type and regimen categorized by the duration since the receipt of the last dose could not be calculated for severity prevention given the small sample size of those with moderate to critical diseases. Lastly, we did not perform analysis for factors correlating with VE or subgroup analysis of VE among populations at-risk for COVID-19 progression due to the limited sample size of both case and control in such population groups.

In conclusion, for preventing Omicron variant-associated infections, the VE increased along with the increase in the number of vaccine dose and the 4-dose regimen (2 boosters) had the highest but only moderate VE. For preventing severity, the three- and four-dose regimens (1 and 2 boosters) had comparable high VE and their effectiveness was more durable than the two-dose regimens. Based on the study findings, current mass vaccination programmes should focus on reducing COVID-19 severity and mandate at least one booster dose after primary series vaccines. The heterologous boosters with viral vector and mRNA vaccines were highly effective and can be used in individuals who had previously received the primary series of inactivated viral vaccines.

## Supplementary Material

Supplemental Material
