# Supplementary material for: Real-life effectiveness of COVID-19 vaccine during the Omicron variant-dominant pandemic: how many booster doses do we need?
Source: Emerg Microbes Infect. 2023 Feb 15;12(1):2174779. doi: 10.1080/22221751.2023.2174779 (PMC9936995; doi:10.1080/22221751.2023.2174779)
Supplement: Supplemental Material [file TEMI_A_2174779_SM5089.doc]

**Supplementary Table: Criteria for patient under investigation (PUI) used in this study**

| **Group** | **Characteristic** |
| --- | --- |
| Persons suspected of having COVID-19 | Having symptoms including fever (temperature of > 37.5 °C), cough, rhinorrhea, loss of smell, loss of taste, rashes, diarrhea or shortness of breath with one of the followings within 14 days prior to the symptoms   - History of travel to or from or stay in an area with reported Covid-19 cases - Working with tourists, in crowded setting or contact with numerous people - Visiting crowded settings, such as markets, department stores, healthcare facilities or public transportation hubs - Contact with confirmed COVID-19 cases - Healthcare workers who contact with suspected or confirmed COVID-19 cases |
|  | Persons with pneumonitis with one of the followings   - No explainable cause or no response to treatment within 48 hours - Severe symptoms or death without explainable cause - Compatible chest imaging results with COVID-19 |
|  | Persons in clusters of cases with respiratory tract infection with negative influenza tests (rapid antigen or polymerase chain reaction test) with one of the followings   - For healthcare workers, the cluster has at least 3 persons in the same working unit and in the same week - For non-healthcare workers, the cluster has at least 5 persons in the same places and in the same week with epidemiological linkage |
| Persons with high-risk contact with confirmed COVID-19 patients | Asymptomatic persons with one of the following risk characteristics   - Having confirmed COVID-19 cases living in the same place - Healthcare workers and other patients who contact with COVID-19 cases without wearing appropriate personal protective equipment - Persons who talk with a COVID-19 case within one meter for more than 5 minutes or stay in the closed space with a COVID-19 case within one meter for more than 15 minutes without wearing masks |
|  | Close contacts in the same vehicle   - Tourists travel in the same group of a COVID-19 case - Airplane passengers sitting in the same row, 2 rows before or 2 rows after the row that a COVID-19case sits in without wearing masks for more than 5 minutes - Coach passengers sitting in the same travel class or unit of a COVID-19 case without wearing masks for more than 5 minutes - Drivers of passenger vehicles which a COVID-19case sits in, except for pilots - Airline crews who serve passengers in the zone where a COVID-19 case sits in |
|  | Close contacts with a COVID-19 case in the same crowed places, such as movie theaters, sport fields, concerts, events, and religious rituals within 1 meter for more than 5 minutes without wearing masks |
